# CTD^2^ Dashboard: a searchable web interface to connect validated results from the Cancer Target Discovery and Development Network

**DOI:** 10.1093/database/bax054

**Published:** 2017-08-08

**Authors:** Bülent Arman Aksoy, Vlado Dančík, Kenneth Smith, Jessica N. Mazerik, Zhou Ji, Benjamin Gross, Olga Nikolova, Nadia Jaber, Andrea Califano, Stuart L. Schreiber, Daniela S. Gerhard, Leandro C. Hermida, Subhashini Jagu, Chris Sander, Aris Floratos, Paul A. Clemons

**Affiliations:** 1 Computational Biology Center, Memorial Sloan Kettering Cancer Center, New York, NY 10065, USA; 2 Chemical Biology and Therapeutics Science Program, Broad Institute of Harvard and MIT, Cambridge, MA 02142, USA; 3 Department of Systems Biology, Columbia University, New York, NY 10032, USA; 4 Office of Cancer Genomics, National Cancer Institute, National Institutes of Health, Bethesda, MD 20892, USA; 5 Computational Biology Program, School of Medicine, Oregon Health and Science University, Portland, OR 97239, USA; 6 Department of Biomedical Informatics, Columbia University, New York, NY 10032, USA

## Abstract

The Cancer Target Discovery and Development (CTD^2^) Network aims to use functional genomics to accelerate the translation of high-throughput and high-content genomic and small-molecule data towards use in precision oncology. As part of this goal, and to share its conclusions with the research community, the Network developed the ‘CTD^2^ Dashboard’ [https://ctd2-dashboard.nci.nih.gov/], which compiles CTD^2^ Network-generated conclusions, termed ‘observations’, associated with experimental entities, collected by its member groups (‘Centers’). Any researcher interested in learning about a given gene, protein, or compound (a ‘subject’) studied by the Network can come to the CTD^2^ Dashboard to quickly and easily find, review, and understand Network-generated experimental results. In particular, the Dashboard allows visitors to connect experiments about the same target, biomarker, etc., carried out by multiple Centers in the Network. The Dashboard’s unique knowledge representation allows information to be compiled around a subject, so as to become greater than the sum of the individual contributions. The CTD^2^ Network has broadly defined levels of validation for evidence (‘Tiers’) pertaining to a particular finding, and the CTD^2^ Dashboard uses these Tiers to indicate the extent to which results have been validated. Researchers can use the Network’s insights and tools to develop a new hypothesis or confirm existing hypotheses, in turn advancing the findings towards clinical applications.

**Database URL:** https://ctd2-dashboard.nci.nih.gov/

## Introduction

The current Cancer Target Discovery and Development (CTD^2^) initiative is a collaborative group of 13 different research teams, or Centers, organized under the auspices of the National Cancer Institute’s Office of Cancer Genomics. It aims to functionally validate discoveries from large-scale adult and pediatric cancer-genome characterization initiatives, and advance them towards use in precision medicine. CTD^2^ Centers use high-throughput experimental and bioinformatic approaches to mine the data and find alterations that potentially influence tumor biology. The Network characterizes the functional roles of candidate alterations in cancers, and identifies novel approaches that target causative alterations through their associated biological pathways. Through the individual and synergistic efforts of the Centers and broad sharing of data with the research community, CTD^2^ contributes to understanding the mechanisms of cancer initiation, maintenance, and progression, and supports the accelerated development of clinically useful markers, targets, and therapeutics.

CTD^2^ is a ‘community resource project’ [https://www.genome.gov/pages/research/wellcomereport0303.pdf] meaning all data are openly available to the scientific community and can be accessed without restrictions. The Network as a whole applies a wide range of investigational approaches, including computational biology, genome-wide loss-of-function or gain-of-function *in vitro* and *ex vivo* screening, high-throughput small-molecule screening, and protein-protein interaction analysis, among others. The web-based open-access CTD^2^ Data Portal [https://ocg.cancer.gov/programs/ctd2/data-portal/] is the primary outlet by which the Network shares these diverse datasets (positive and negative results generated as part of each Center’s research) and the associated project descriptions and methodologies. Although the Network has a dedicated process to harmonize the data, a certain level of bioinformatics expertise is needed to use the data in the Data Portal to their fullest potential. The data in the Portal are generated with technology-relevant controls and undergo quality assessment, but have not been independently validated [https://ocg.cancer.gov/sites/default/files/CTD2CaveatEmptor_final.pdf]. Therefore, researchers who are using the data must further validate findings in order to determine the significance and strength of a particular result. Network results are shared through published manuscripts, which contain data and related information. However, these data are not always provided in an easily digestible format for all types of researcher. Such publications concentrate on the biological relevance of a few findings the authors find interesting, although in large-scale high-throughput experiments, there may be many other positive findings.

The CTD^2^ Dashboard [https://ctd2-dashboard.nci.nih.gov/] addresses these data-access and usability challenges and fills the need for a searchable and browsable web interface that assembles concisely summarized results, connects them with subsequent evidence that reinforces or builds upon the original finding, and conveys the extent to which the results have been functionally validated. The Dashboard was developed to address the need of the community to find data generated by CTD^2^, and to adhere to the FAIR (findable, accessible, interope rable and re-usable) principles (1). The Dashboard was designed to allow easy navigation and use by a range of scientists, including both computationalists and non-computational cancer experts.

## Database structure and organization

### The Dashboard concept

The CTD^2^ ‘Dashboard’ (which was developed by Centers at the Broad Institute, Cold Spring Harbor Laboratories/Memorial Sloan Kettering Cancer Center, and Columbia University, with input from the entire CTD^2^ Network) compiles CTD^2^ Network-generated conclusions, termed ‘observations’, associated with particular experimental subjects. Any researcher interested in learning about a particular gene, protein, or compound that has been the subject of experimentation by Network members can come to the CTD^2^ Dashboard to quickly and easily find, review, and download Network-generated results. In particular, the CTD^2^ Dashboard allows users rapidly to connect different cancer biology experiments about the same biological entity, including its function in the experiment, carried out by multiple Centers in the Network.

**Figure 1.**
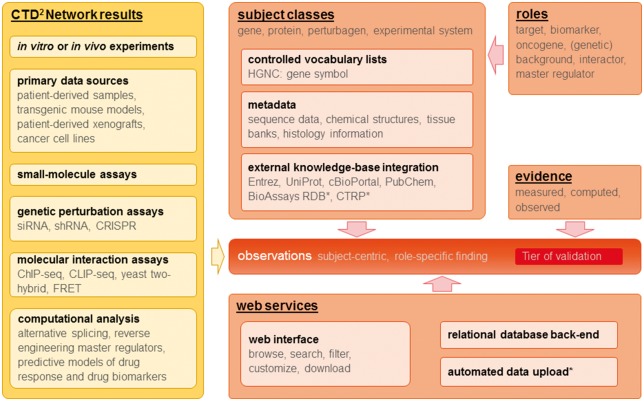
Overview of CTD^2^ Dashboard data types, key concepts, and web services. Items marked with (_*_) represent work in progress or future plans.

Because of the diversity of data types generated by the contributing Centers, the concept of the CTD^2^ Dashboard needed to be very flexible. Indeed, one of the early conceptual challenges was to provide for a situation where the types of experiments to be performed were not known in advance. As described in detail in later sections, we addressed this challenge by defining a small number of subjects that represent actual biological entities (genes, proteins, etc.), but did not pre-define the types of data that could be connected. Instead, we articulated a system of evidence that is defined in terms of *electronic* data types (e.g. numbers, text labels, images, URLs, etc.). This strategy enables the CTD^2^ Dashboard to connect observations from diverse types of experiments and analyses to represented subjects (Figure 1).

To distinguish the *strength* of different types of evidence, the CTD^2^ Network has developed broadly defined Tiers of validation for evidence pertaining to a particular finding (2). Specifically, Tier 1 evidence comprises high-throughput biology, such as screening or profiling experiments, unbiased or genome-wide computational analyses, and other similar hypothesis-generating activities. Evidence is classified as Tier 2 when an initial hypothesis is validated *in vitro*, such as with protein-binding assays or cell-culture models. Tier 3 evidence is supported by validation of a hypothesis *in vivo*, such as in mice, organoids, or other models. Through the CTD^2^ Dashboard and application of the Tier ranking system, any investigator can utilize the Network-generated experimental results as a starting point to extend or confirm hypotheses.

### Observations–atomic units of the CTD^2^ Dashboard

The main challenge in developing the CTD^2^ Dashboard was devising a data model capable of representing datasets generated by the large and diverse collection of computational and experimental assays performed by the CTD^2^ Network Centers (Table 1). Our solution was to organize data around the concept of an *observation*. Observations are statements about biological or chemical entities (e.g., genes, compounds, cell lines, etc.) that concisely summarize experimental results. As described above, CTD^2^ Dashboard observations are annotated with Tiers based on the strength of the supporting evidence. The highest, Tier 3, is assigned to observations representing hypotheses validated *in vivo*. Typically, these observations correspond to research findings reported in the literature and are the most comprehensively validated results generated from analysis of Network data. For example, a high-throughput screen designed to assess how different genetic backgrounds influence response to therapeutics could give rise to the following observation: ‘Across many cancer types, cell lines *sensitive* to *BIX-01294* are enriched in cells harboring *LYN* mutations’. The italicized words in this example highlight the building blocks of an observation, namely the subjects, and are hyperlinked in the Dashboard interface. The bold text ‘sensitive’ summarizes a piece of evidence that defines the result (enrichment among sensitive cell lines, in this case). Additionally, observations are linked to background information and other evidence, i.e., assay-specific data which corroborate the observation statement. For instance, for the example observation shown above, relevant evidence could include the false discovery rate, an enrichment plot and the number of cell lines used. These CTD^2^ Dashboard principles are illustrated in more detail in subsequent sections.

**Table 1.** Overview of CTD^2^ Dashboard datasets

High-throughput or bioinformatic method	# Tier 1 observations (May 2017)
Cancer cell-line sensitivity profiling (9)	10 828
Oncogenomic screening – average tumor volumes (18)	18
Master-regulator analyses (17,28)	38
shRNA screens and ATARiS (19,23)	6961
siRNA functional kinomics screen (21)	58
MethylMix analysis (25)	58
Elastic-net analysis of TCGA (27)	7176
Evaluation of dependency differentiality (EDDY) analysis (20)	164
Chemical-genetic interaction mapping (24)	156
Quantitative-trait locus variant identification (26)	19
Drug-sensitivity screening (22)	66
Reverse phase protein arrays (22)	24

Tier 1 observations associated with unique CTD^2^ Dashboard ‘projects’ (9,17–28) are listed for each high-throughput assay or bioinformatics approach, as of May 2017.

### Subjects–focus of the experiments

As one mission of the CTD^2^ Network is to discover and develop new therapeutic hypotheses, we expected that cancer-relevant genes and the proteins they encode (e.g., oncoproteins) would need to be explicit in our model. In addition, perturbation reagents such as small molecules or shRNAs, and certain common experimental systems, such as named cell lines and mouse models, were also included. ‘Subjects’ are the focus of experiments and are categorized into classes, such as genes, proteins, perturbagens, and model systems. A critical first decision in conceptualizing the CTD^2^ Dashboard was to decide which entities would be considered well-defined classes for the purposes of cross-linking different types of evidence. Together, these subject classes form the core knowledge skeleton of the CTD^2^ Dashboard, allowing researchers from multiple Centers to connect observations and other evidence to the same abstract entity, even when studied in separate laboratories. These subject names are made consistent through vocabulary control and synonym reconciliation, ideally *via* an external authority (e.g., gene names and synonyms from HUGO Gene Nomenclature Committee database). In some cases, such as for cell lines and mouse models, no suitable external authority was already available, so the CTD^2^ Dashboard development team curated and reconciled synonyms for these subjects. This construction allows the knowledge built around a subject to be greater than the sum of the individual contributions.

Subject choices permit extensive connection to structured resources outside of the CTD^2^ Dashboard for additional external data and metadata. Importantly, each subject class warrants connection to different types of metadata and resources. For example, genes and proteins are described with controlled names and identifiers, and the CTD^2^ Dashboard links out to other resources, including Entrez and UniProt, which provide additional summary information, genomic context, functional annotations, macromolecule sequences, and references. Genes are also linked to their monograph pages in the cBioPortal (3,4), which provides frequencies of alteration of the gene in different cancer types, as well as mutation and expression-level information in collections of samples.

Similarly, small-molecule probes and drugs can connect to sources of outside information, as many public databases of such entities are available (5–7). At present, small-molecule monographs in the Dashboard link to their corresponding entries in PubChem (7), which provide summaries of activity and mechanism of action, where available, along with chemical information and descriptors, structure representations, and links to bioactivity assays using the compound. New versions of the Dashboard will add links to other resources, such as the BioAssay Research Database (8) and the Cancer Therapeutics Response Portal (9–11), through which additional information about compound activity, including in cancer cell models, can be accessed. By linking to external data about subjects whenever possible, we enrich the CTD^2^ Dashboard with content beyond that generated explicitly by the CTD^2^ Network.

### Roles–functions assigned to subjects based on supporting data

Due to the diversity of experiments done by CTD^2^ Network Centers and the inherent characteristic of subjects to have multiple possible biological functions, subject classes alone are not sufficient to provide all the contextual information needed to understand the relationships between subjects. We introduced additional functional classifications for subjects, termed ‘roles’, which are assigned to each subject in a CTD^2^ Dashboard submission to delineate how it is acting or being used in an experiment, and to help interpret the associated evidence. In general, roles are standardized and new terms are added to the list of potential roles only after careful consideration. This strategy prevents submitters from assigning very specific and unique roles to each use of a subject, which would render the use of roles as a grouping mechanism less effective. In the Dashboard, roles for genes and proteins include target, biomarker, (genetic) background, interactor, (candidate) master regulator, and oncogene. In the Dashboard interface, a focus on targets and biomarkers is selected by default, but users can add or remove roles in the Browse interface using a check-box list under the button ‘Select Roles’. For compounds and other perturbagens, the roles of perturbagen and (candidate) drug are default, with an additional role of control compound available. Finally, cell and tissue samples associated with a particular disease context have roles of disease, metastasis, and tissue (of origin). Roles thus allow for easier browsing and searching.

### Evidence–data supporting an observation

While subjects must conform to their type-specific controlled vocabularies, submitters are allowed complete flexibility when providing evidence pertinent to an observation. This information can include numeric values or text displayed directly on observation pages, files (including images) that are served by the CTD^2^ Dashboard’s web-server, or URL hyperlinks to other websites. Thus, CTD^2^ Dashboard evidence is constrained and categorized not by its biological meaning or context, but rather by its electronic data type. Furthermore, to indicate *how* a particular piece of evidence was obtained, we categorize them as ‘observed’, ‘measured’, or ‘computed’. Again, these classifications are more general than any particular biological context or interpretation, and together these broad evidence types and categories encourage submitters to define evidence in new and creative ways. Each of the existing Dashboard evidence types and roles are mapped to their corresponding Evidence Ontology (http://www.evidenceontology.org/) codes and appropriate hyperlinks are provided to the corresponding Evidence Ontology pages. Furthermore, we encourage submitters to use Evidence Ontology terms in their submissions to provide further resolution of classes of evidence.

## Data submissions and curation

Data submitted to the Dashboard by CTD^2^ Network Centers can be accessed through the web interface [https://ctd2-dashboard.nci.nih.gov/]. The primary goal of the interface is to enable inter-connecting, browsing, searching, and presentation of the CTD^2^ Dashboard data through a front-end that is intuitive to use and accessible to scientists at all levels of bioinformatics expertise. A key element that enables this functionality is the consistent use of controlled subject classes across all Center submissions. These serve as unambiguous identifiers that facilitate cross-referencing of data from different Centers to identify observations referring to the same abstract biological entities.

The CTD^2^ Dashboard is geared towards sharing observations with scientists and others not specializing in computational biomedicine (Figure 1). New content is contributed to the CTD^2^ Dashboard through individual ‘submissions’ by the CTD^2^ Network Centers. A submission is a collection of observations that were obtained from one investigation, possibly integrating more than one type of experiment, and whose results share the same structure and format. For functional biology experiments (Tiers 2 and 3), submissions often consist of only a single observation. Currently the process of preparing, curating, and uploading submissions is manual. Submitters, with the help of the CTD^2^ Dashboard team, identify fields (subjects, background information, and evidence) pertinent to observations, assign classes, roles, and types to the fields, and provide, in a tabular file format, descriptive text for each field displayed on the corresponding observation page. A submission must contain an ‘observation summary’ that describes each observation and is displayed on browse and search pages. These summaries use variables (i.e., subject classes that are ‘filled in’ by the submitting Center) and additional text to provide context for interlinking subjects appropriately. Multiple observations of the same type (e.g., describing multiple results from a certain experiment or analysis), but about different subjects of the same class, can be handled by a single observation summary. Prior to loading to the CTD^2^ Dashboard, submissions are automatically checked for internal consistency and for consistency with current CTD^2^ Dashboard content. Recently, the CTD^2^ Dashboard team created a web-based submission-generation system to guide contributors through the process. To manage quality control, at this time only CTD^2^ Network Centers can contribute new submissions to the Dashboard. However, we envision that this system may open the door for future Dashboard submissions from outside the CTD^2^ Network. To keep users up-to-date on the latest additions to the Dashboard, an RSS feed feature is provided, which allows subscribtion to receive a notification of submissions or observations involving a subject of interest (e.g., gene, protein, or compound).

### Contextual architecture is independent of subject matter

The fundamental structure of the CTD^2^ Dashboard architecture is not coupled to cancer targets, cancer biology, or even biology. Indeed, any system of controlled subject names could serve as a knowledge skeleton for attaching evidence. We have not fully explored alternative knowledge domains beyond the conceptual stage, but certainly encourage others to do so. Controlled-name subjects with any entity-relational framework could be coupled with evidence classes used in the current CTD^2^ Dashboard: labels, numeric values, URLs, images, and attached files. In this sense, the CTD^2^ Dashboard serves as a way to link an ‘operating system’ of file types with each member subject of a given class. In other words, the Dashboard does not need to understand the contents of the evidence being linked, only the format in which that evidence is being presented.

## User interface

The end-user-facing web application is constructed in two layers. The back-end is implemented as a series of RESTful web services based on the Spring framework, a widely used application framework and inversion-of-control container for the Java platform [https://spring.io/guides/gs/rest-service/]. The front-end is developed using the lightweight backbone.js JavaScript library [http://backbonejs.org/docs/backbone.html]. The design provides a loose and flexible coupling between the two layers, allowing for each part to be developed and maintained independently from the other. The database supporting the CTD^2^ Dashboard web interface is built using the Hibernate implementation of the Java Persistence API, which is seamlessly supported by the Spring framework. The back-end RDBMS used is MySQL. The CTD^2^ Dashboard source code is open and publicly available on GitHub [https://github.com/CBIIT/nci-ctd2-dashboard/].

Researchers who visit the CTD^2^ Dashboard to investigate a specific gene or compound will find the Search function to be the most expedient method for locating relevant data. CTD^2^ Dashboard data are indexed against subject terms and can be queried using the search box located in the top menu bar of the CTD^2^ Dashboard. For instance, searching for a full or partial gene symbol will generate a list of all matching genes along with a count of the observations involving each gene; each count is hyperlinked to a detailed list of the relevant observations.

In addition to search, data can be explored *via* several browsing options.

**Figure 2.**
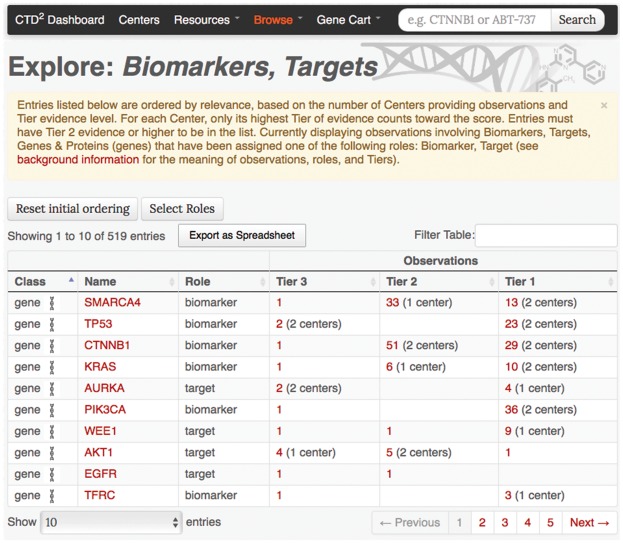
‘Biomarkers, Targets, Genes & Proteins’ browse page. Every table row corresponds to a gene–role pair, and displays for each evidence Tier the number of observations where the gene has been reported to play the specific role. The number of Centers who have contributed towards the observations is also shown. The ‘Select Roles’ button can be used to customize this view by adding or removing roles beyond those used in the default listing (‘biomarker’ and ‘target’). The initial ordering of the table rows is determined by a relevance score calculated as the sum across Centers of the highest Tier evidence provided by each Center contributing evidence.

- *Subject-based browsing*: Exploration of CTD^2^ Dashboard entries can be initiated from a summary view. The CTD^2^ Dashboard offers three “Browse” pages focusing on genes, compounds, and cancer types. Each page provides a list of biological entities prioritized according to the number, breadth, and level of validation of the observations involving those entities. For example, the ‘Biomarkers, Targets, Genes & Proteins’ browse page focuses on genes and lists roles and observations associated with each subject (Figure 2). Users can download this table as an Excel file using the function called “Export as Spreadsheet”.
- *Submission-based browsing*: The ‘Centers’ link at the site’s top navigation bar leads to a page that lists all the CTD^2^ Network Centers. From that list, users can access all data submissions produced by a Center and drill down to the observation data in each submission. There is also a link which allows the user to easily and directly download the entire submission source data package.
- *Story-based browsing*: Research findings that have been explored in detail are featured prominently on the homepage of the CTD^2^ Dashboard in the form of stories. The aim of stories is to distill the essential elements of reported results, including integration of many experiments at different levels of evidence, at a level of detail that is appropriate for a general scientific audience.

**Figure 3.**
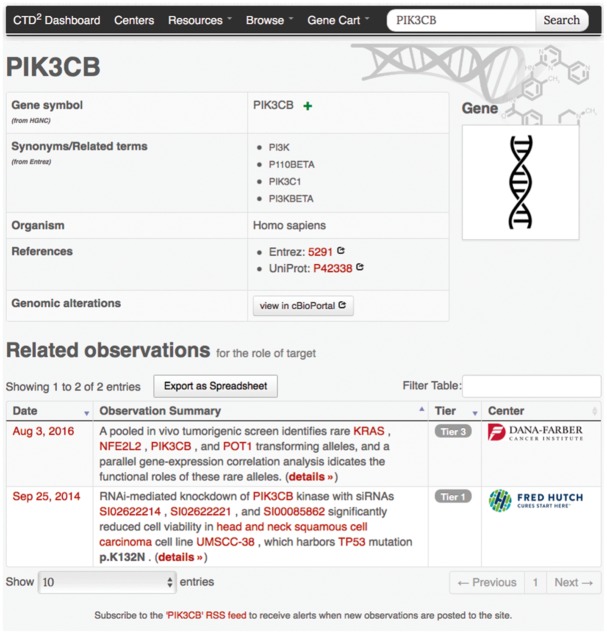
Example gene-profile page. Gene-profile page for *PIK3CB* shows synonyms (as defined by Entrez), provides links to external annotation web sites, and lists observations involving *PIK3CB* along with their Tiers and the submitting Center names. Similar detail pages are available for all subject types supported by the CTD^2^ Dashboard.

**Figure 4.**
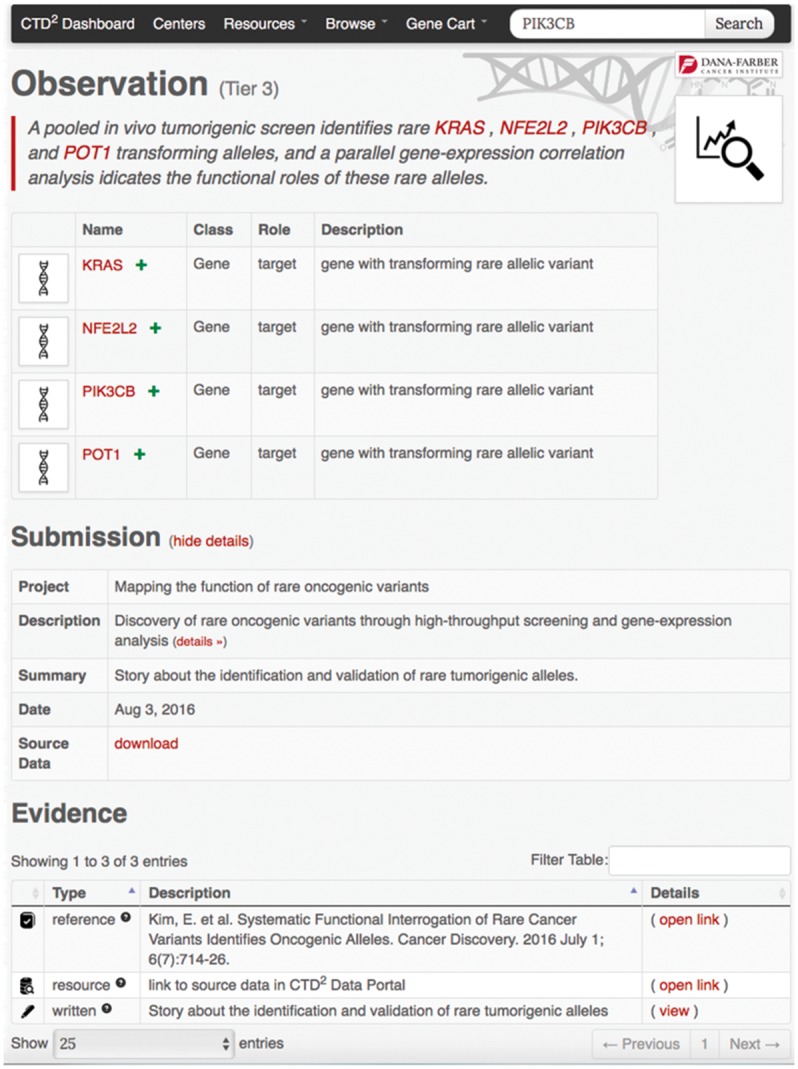
Example observation page. This page comprises an observation summary, a list of implicated subjects, a download link to information about the data submission containing the observation, and the evidence supporting the observation findings.

Search and browse operations ultimately result in a list of entry points to detailed information about observations and subjects. For instance, clicking on any gene symbol under the ‘Name’ column in the gene-browser list leads to a gene profile page (Figure 3) where the top portion contains key gene descriptors and links to relevant annotations and resources, and the bottom portion lists the observations generated by the Centers involving that gene. As with the browse feature, users can download this entire table as an Excel file using the button ‘Export to Spreadsheet’. Similarly, clicking on the ‘details’ link of any observation brings up a page (Figure 4) describing the observation attributes, including:

- a short summary of the related investigational finding (i.e., the *observation summary*);
- the biological entities (genes, compounds, cell lines, etc.) involved in the study and their specific roles;
- description of the submission, submission date, and a link to download all the data (evidence) associated with that submission;
- experimentally and computationally derived evidence supporting the reported finding.

### Gene Cart

**Figure 5.**
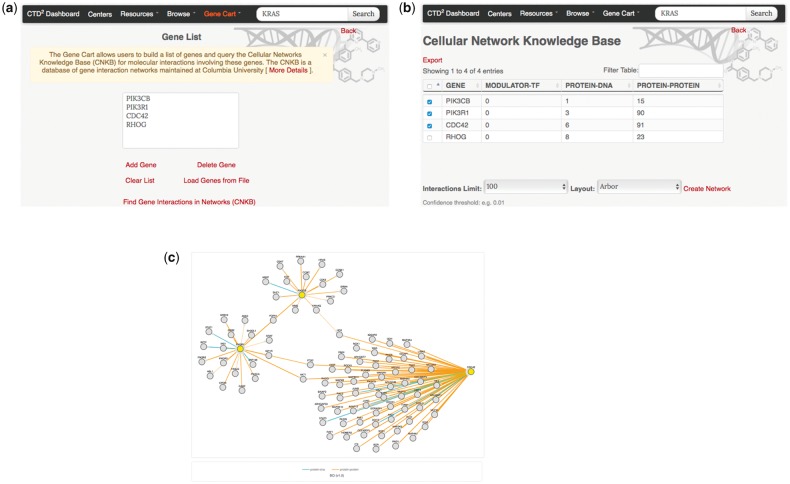
The CTD^2^ Dashboard Gene Cart feature. (a) The ‘Gene Cart’ link available on the top navigation bar leads to a page that lists all genes that have been added by the user. From this page, users can add or remove genes from the list and initiate a query of the Cellular Networks Knowledge Base. (b) After selecting interactome type and version (in this example, B-Cell Interactome v1.0 was chosen) query results are summarized in a tabular view, with one row per query gene. Each row shows the number of interactions involving the corresponding gene, categorized according to interaction type (protein–DNA or protein–protein). Results can be exported as a SIF format file for display in third-party software such as Cytoscape (29,30). (c) Users can also select one or more of the query genes and click on the ‘Create Network’ link to generate a graph view of the interactions. Nodes in yellow represent query genes. Edges represent interactions and are colored according to the type of interaction.

Many CTD^2^ Dashboard pages contain green ‘+’ icons adjacent to gene symbols (see Figures 3 and 4). During Dashboard data exploration, users can click on these icons to incrementally compile a list of genes of interest. This list can be accessed through the ‘Gene Cart’ link available in CTD^2^ Dashboard’s navigation menu (Figure 5a). From there, users are able to edit the contents of the list and to query the Cellular Networks Knowledge Base (CNKB) (12) repository of molecular interaction networks to retrieve interactions involving the genes in the Gene Cart (Figure 5b and c). CNKB (12) is a database of protein and gene interactions maintained at Columbia University. It comprises curated pathway collections and cancer tissue-specific regulatory and signaling networks derived computationally through the analysis of large gene-expression datasets (13) such as those generated by The Cancer Genome Atlas, and also predicted or measured protein-protein interactions (14,15). The ability to interrogate CNKB and to explore the interactions involving key tumor regulators can help users better contextualize and interpret Dashboard observations. For instance, if an shRNA screen reduces the viability of a tumor cell line, looking at the interaction network involving the shRNA targets may reveal the underlying mechanism of action by identifying genes regulated by these targets.

## Discussion

As content continues to grow and submitters continue to find creative uses for the Dashboard as a data-sharing platform, the CTD^2^ Dashboard team will continue to improve the database and interface (Figure 1). Our current major undertaking to improve CTD^2^ Dashboard functionality is creating a web-based submission-generation system that uses standardized formats, thereby reducing the time and complexity of the submission process by automating data import and validation. Plans to improve the user experience and further enrich the content include linking to additional outside resources that the Network selects as useful. For example, on the gene-entry pages, hyperlinks will point users to GeneCards and HGNC. The ‘Compounds & Perturbagens’ browse pages will include links to ChemBank (6,16), BioAssay Research Database (8), and ZINC (5). Links to relevant cell lines at American Type Culture Collection (ATCC) will be added to the tissue-type entries. In keeping with the FAIR principles (1), further evolution of the Dashboard will continue to expand the use of cross-links with other community resources.

As CTD^2^ Dashboard content grows, evidence to support higher-Tier rankings increases, and additional external resources are integrated, the opportunities for its use will expand. Already, many Dashboard submissions about the same subjects are interconnected across multiple Tiers, reflecting the evolving validation within CTD^2^ Network projects. The wealth of information embedded in the CTD^2^ Dashboard can be taken advantage of to confirm hypotheses and initiate new collaborations. We plan to take advantage of this information to explore new bioinformatic analyses that may yield new cancer dependencies. We encourage the research community to use this database to study the experimental approaches the Network has taken, explore the data using an easily minable format, develop new hypotheses, and build on CTD^2^ Network findings.
